# Embracing the principles of practice transfer to get the word out on the new metabolic dysfunction-associated steatotic liver disease nomenclature

**DOI:** 10.1097/HC9.0000000000000275

**Published:** 2023-09-27

**Authors:** Rotonya M. Carr

**Affiliations:** *University of Washington, Department of Medicine, Division of Gastroenterology Seattle, WA*

## INTRODUCTION

NAFLD has a new name, metabolic dysfunction-associated steatotic liver disease (MASLD) (pronounced ma-zuld). This new moniker was announced at the European Association for the Study of Liver Diseases (EASL) conference on June 24, 2023. MASLD is now one subcategory of steatotic liver disease, an overarching categorization of diseases that cause an accumulation of fat in liver cells.^1^ While there is global *endorsement* of this change in nomenclature by professional and hepatology patient advocacy organizations, we must ensure global *adoption* by immediately turning our attention to the broader community who are likely unaware of this news.

Having been involved in the Delphi process that led to the name change of NAFLD,^1^ I must admit that I was ill-prepared to help promote adoption of the new nomenclature within my organization and local community. Up until now, my efforts have been rather haphazard, lacking a systematic approach to reaching the audiences most impacted by these changes.

To provide some context, my methods so far have included informing patients as they entered my clinic; creating a personal “dot phrase” shortcut for our hospital’s electronic medical record system to notify referring doctors of the change (“NAFLD, now called MASLD …”); sharing the announcement with colleagues in my division and personal networks; and leveraging social media platforms to amplify the nomenclature. I want to emphasize that I do not dismiss the significance of these attempts; rather, I acknowledge that they lacked a strategic approach and were mostly “one-offs.”

## PRACTICE TRANSFER

Fortunately, both myself and other members of the community of providers and advocacy groups can draw upon the wealth of literature on practice transfer, or the dissemination, acceptance, and adoption of new ideas within organizations. Although most of the practice transfer literature involves new methodologies or clinical practices, we can still apply at least some of this guidance to adoption of nomenclature changes. Based on their retrospective qualitative study of 13 Kaiser Permanente transfers, Tallman et al^2^ summarized that the likelihood of successful practice transfer hinges not only on factors relevant to the leaders who promote change but also on factors relevant to the recipients. Namely, recipients need to feel there is a compelling problem to solve; accept that there is evidence supporting the superiority of the new practice; trust and have clear communications with the leaders and “source champions” (practice experts or innovators who help communicate the message); and be able to observe the success of the new model in practice. Relying solely on evidence is rarely sufficient to motivate recipients to adopt changes in practice. Practice transfer is also more successful when there is leadership support, adequate resources to support the change, and a culture that is open to change. Consequently, some strategies that can overcome barriers to practice transfer include demonstration that the practice addresses a high-priority issue, organizing a multidisciplinary team to plan the implementation strategy, enlisting leaders and source champions who believe in and can communicate the change, and engaging leaders to ensure that sufficient resources are allocated for practice transfer.

These suggestions were further distilled into the “4 A’s” by King et al^3^: awareness, assessment, alignment, and action, themes that reflect the technical and social components of change. The first step, awareness, involves communicating information in one’s facility or region that “a better practice exists.” Following awareness, the next step is to assess the likelihood of acceptance of the proposed practice. This assessment should involve asking specific readiness questions, such as whether a small pilot could be trialed first, whether there are clear advantages adopting the proposed practice over the current one, whether the change will work in the given culture, whether the practice can be observed in action, and what level of disruption will the change cause.

Once the assessment is complete, the focus should shift to alignment and support. It is critical to ensure key stakeholders are aligned with and supportive of the change. This is largely achieved through effective communication and mitigating barriers to adoption. Action is the final step in implementing the practice. A phased-in, measured approach allows time for both trial and observation. Specific strategies that address the 4 A’s are found in Table 1.

**TABLE 1:** Adapted from the 4 A’s^3^ of practice transfer^3^

Awareness	Assessment	Alignment	Action
Convene conferences/meetings Establish peer networks Draft and disseminate documents Publish in journals/websites Hold briefings Facilitate direct follow-up communications with the source champion	Assess trialability Assess advantage Assess culture compatibility Assess observability Assess simplicity	Identify a sponsor, physician champion, and lead implementor Form a multidisciplinary planning/ steering committee of influential stakeholders	Use a phased-in approach Document progress

Although the Delphi Conference and the subsequent communication strategy for clinicians and patient advocacy groups seem to have marched in lock-step with the guidance on practice transfer, there is still an opportunity for us to extend these principles to the global community of patients, clinicians, and general public. In so doing, we must be prepared for a slower adoption of the new nomenclature among nonhepatologist physicians and the lay public, despite such a declarative change. The pace of this “diffusion of innovation” as Rogers initially described^4^ and further elaborated upon by Berwick^5^ is largely influenced by recipient buy-in. In essence, recipients are likely to ask 3 key questions: (1) Why is this change important? (2) What are the benefits for me? (3) How much inconvenience will implementing this change cause?

On average, the pace of practice transfer follows an S-shaped curve, where exponential growth in adoption occurs only after reaching a “tipping point” threshold of at least 10%-20% early adopters, with 50% adopting new ideas later in the process.^4^ The whole process can take up to 27 months!^2^ Indeed, in recent hepatology nomenclature history, the name change from Primary Biliary Cirrhosis to Primary Biliary Cholangitis took ~2 years from the official change in nomenclature by the American Association for the Study of Liver Diseases (AASLD) and EASL in 2015 to the World Health Organization’s (WHO’s) approval in late 2016 and launch of the nomenclature change promotion campaign by patient groups in 2017.^6^

## STRATEGY

Armed with the understanding of practice transfer principles, I now feel better equipped to help develop a strategy for disseminating the news about the new nomenclature and supporting its adoption within my own institution and community. By understanding the factors that influence recipient buy-in, one can exploit opportunities to influence the pace of diffusion and increase the likelihood of adoption of the new nomenclature. Here are some actionable steps one can take.

Ten steps to promote widespread adoption of the new MASLD nomenclature:

1. Get started: Take the initiative and begin planning at the local level now.
2. Become fluent in the new nomenclature and develop a straightforward way to explain it. Use it in clinical documentation and when talking about the disease to patients and others. Focus on the “why,” namely the new MASLD nomenclature reduces stigma and elevates biology.
3. Engage your health system partners and form a multidisciplinary team of individuals who can serve as nomenclature champions. This team should include clinicians who care for patients with MASLD, patients, and patient advocates. Share contact information of nomenclature champions to facilitate direct communication.
4. Devise an iterative and multimodal plan. Options include written documents, electronic communication, institutional and community talks, video, local news media, and social media. All modalities should use simple language and be disseminated at predefined, well-paced intervals.
5. Make using the new nomenclature easy in charting. Create shortcuts to help replace the old “NAFLD” with the new “MASLD.”
6. Allocate resources to print flyers, posters, or handouts for clinics. Connect others with web-based updates.
7. Participate in opportunities to advocate for early WHO and federal agencies’ adoption.
8. Measure the impact of these efforts (eg, surveys assessing familiarity with the new nomenclature).
9. Share lessons learned and best practices with the global community of professionals.
10. Exercise patience!—with the community, colleagues, and oneself; and engage patients in a meaningful way.

My hope is that by following these steps and reflecting on the science of practice transfer, we all can be more strategic in helping get the word out about the new MASLD nomenclature to our local communities and ease the transition as much as possible for those most impacted by this change.
